# Pleural epithelioid hemangioendothelioma in a 39-Year-old female: a case report

**DOI:** 10.1186/s13019-024-02602-4

**Published:** 2024-03-12

**Authors:** Parviz Mardani, Reza Shahriarirad, Mohammad Nekooeian, Mohammad Hossein Anbardar, Bizhan Ziaian, Hooman Kamran, Nazanin Ayare, Masoud Vafabin, Damoun Fouladi

**Affiliations:** 1 Thoracic and Vascular Surgery Research Center, Shiraz University of Medical Sciences, Shiraz, Iran; 2 School of Medicine, Shiraz University of Medical Sciences, Shiraz, Iran; 3 Health and System Research center, Shiraz University of Medical Sciences, Shiraz, Iran; 4 Shiraz Nephro-Urology Research Center, Shiraz University of Medical Sciences, Shiraz, Iran; 5 Department of Pathology, Shiraz Transplant Center, Abu Ali Sina Hospital, Shiraz University of Medical Sciences, Shiraz, Iran; 6 Students Research Committee, School of Medicine, Shiraz University of Medical Sciences, Shiraz, Iran

**Keywords:** Epithelioid hemangioendothelioma, Pleural tumors, Vascular tumors, Thoracic cancer

## Abstract

**Background:**

Epithelioid hemangioendothelioma (EHE) is a rare malignancy of vascular origin which can be primarily be seen in various tissues. EHE originating from the pleura is an even more uncommon subtype which may mimic mesothelioma and pleural carcinomatosis. The prognosis of pleural EHE is poor and there is no consensus on the optimal therapeutic approach.

**Case presentation:**

A 39-year-old middle-eastern female presented with progressive dyspnea and left shoulder discomfort. Chest computed tomography scan revealed a left side pleural effusion and pleural thickening. Pleuroscopy was done and biopsies were taken which were positive for CD31, CD34, CK, factor 8-R-antigen, and vimentin. Patient was diagnosed with pleural epithelioid hemangioendothelioma (PEHE) and chemotherapy was started and underwent extrapleural pneumonectomy 7 months later. Unfortunately, the patient passed away 10 months after diagnosis due to disease complications.

**Conclusions:**

Once PEHE is suspected in histology it can be confirmed with immunohistochemistry. Chemotherapy, surgery or a combination of both is currently used as the treatment but the standard treatment remains a question.

## Introduction

Epithelioid hemangioendothelioma (EHE) is a rare malignant vascular tumor with an unpredictable prognosis [1]. The clinical course of the disease can vary from a benign hemangioma to angiosarcoma [2]. EHE can arise in many sites but most commonly occurs in the soft tissues, lungs, liver, and bone [3].

EHE primarily originating from the pleura, unlike the lung, is very rare but it is more aggressive than the other types and only approximately 60 cases have been described so far [4–6]. In most of the cases, it presents clinically with pleural effusions and thickening which are unspecific [6]. Diagnosis is usually confirmed by thoracoscopy and immunohistochemistry, but pleural fluid cytology is often not conclusive [7].

In this report, we describe a middle-aged female patient who presented with progressive dyspnea. Pleuroscopic biopsy and histopathological evaluation yielded a diagnosis of PEHE and she subsequently underwent chemotherapy and surgery.

## Case presentation

In January 2021, a 39-year-old middle-eastern female presented to our hospital with a one-month history of increasing dyspnea and left shoulder discomfort. Her dyspnea aggravated with sleep, but was not accompanied by fever, chest discomfort, nausea, vomiting, or weight loss. Except for a prior rhinoplasty, the patient’s previous medical history was insignificant. In addition, there was no history of smoking or cancer in the family. The patient’s vital signs and oxygen saturation were normal upon initial evaluation. Lung auscultation indicated diminished breathing sounds at the base of the left lung. The remainder of the physical examinations were normal. The initial laboratory examinations demonstrated an increase in white blood cell (WBC) count (19.9 × 10^9^/L), hemoglobin level of 10.9 g/dl, platelet count of 521 × 10^9^/L, blood urea nitrate level of 10 mg/dl, creatinine of 0.8 mg/dl, blood sugar of 120 mg/dl, sodium of 141 mEq/L, and potassium of 4.3 mEq/L.

**Fig. 1:**
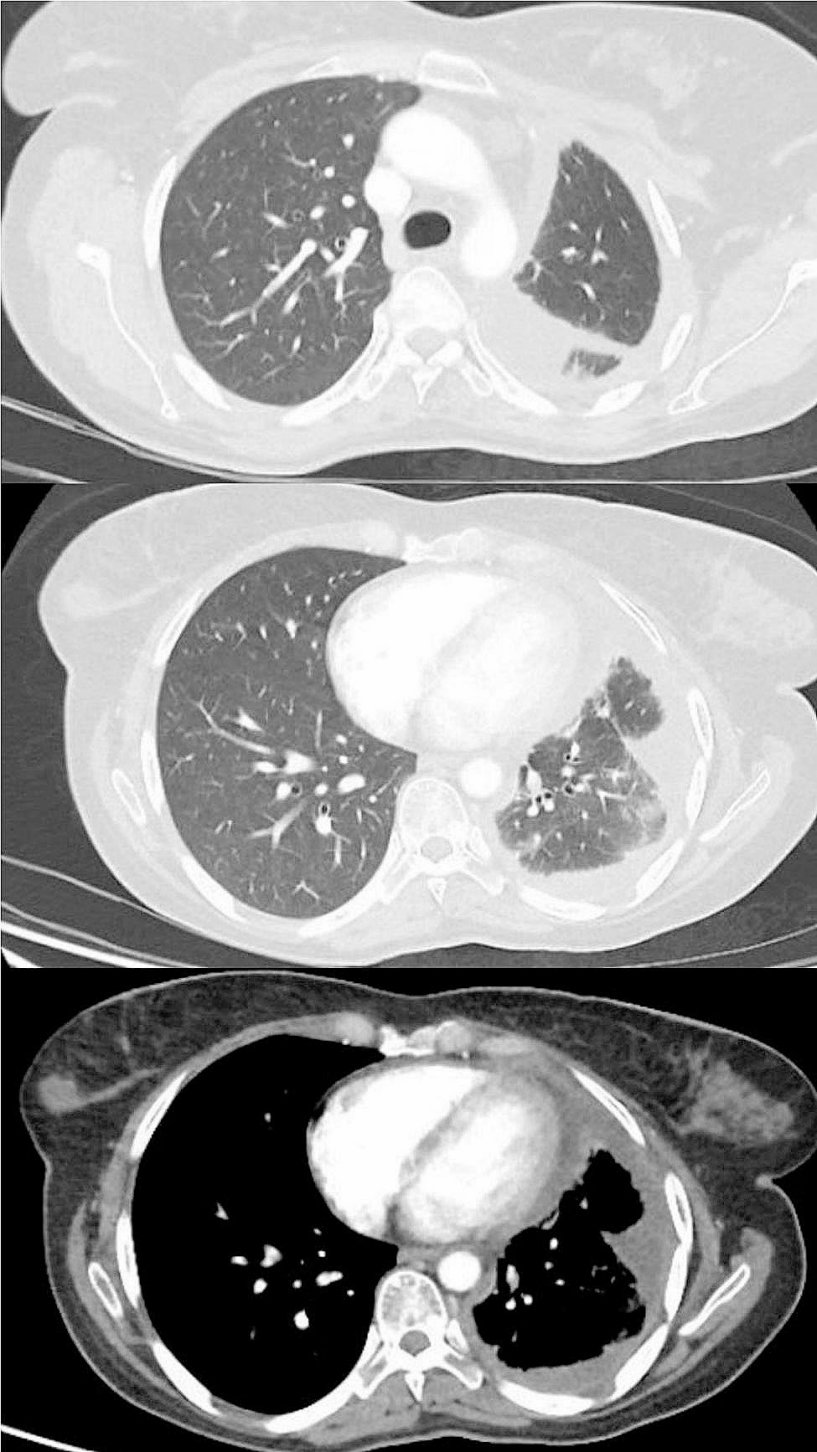
Computed tomography scan of lungs demonstrating left-sided pleural effusion, and minimal pleural thickening, without accompanying parenchymal abnormalities

Chest x-ray revealed opacification of the left lung and computed tomography (CT) scan of the chest showed left-sided pleural effusion, and minimal pleural thickening, without accompanying parenchymal abnormalities (Fig. 1). Electrocardiography and echocardiogram showed no cardiac abnormalities. Subsequently, the patient underwent therapeutic and diagnostic thoracentesis. The findings of the pleural fluid analysis demonstrated a total count of 3100 cells with presence of malignant cells and red blood cell count (RBC) of 2400, WBC count of 700 (lymphocyte 40% and neutrophil 60%), fluid lactate dehydrogenase 299 IU/L, negative adenosine deaminase and culture.

For further investigation of the malignant cells seen in cytology analysis, pleuroscopy was done by a pulmonologist, and multiple creamy nodular lesions were found on the visceral and parietal pleura. Multiple biopsies were taken, pleural effusion was drained, and a chest tube was inserted. After chest tube removal, the patient was discharged and instructed to visit the pulmonologist whenever the biopsy results were ready; However, five days later, the patient was readmitted to our hospital due to development of dyspnea with accompanying chest pain.

**Fig. 2:**
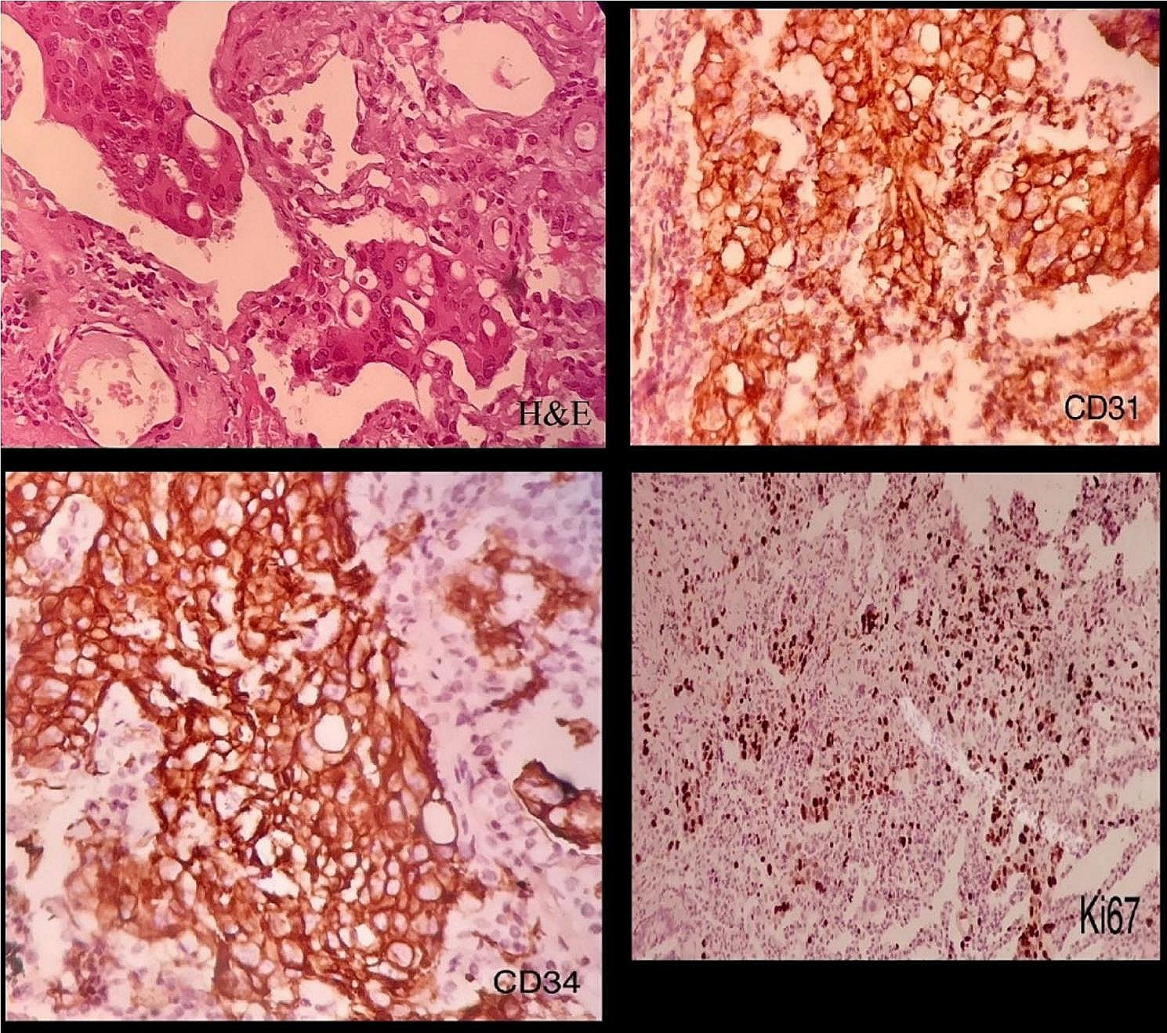
Histopathological evaluation of pleural epithelioid hemangioendothelioma. Microscopic sections show solid nests of epithelioid cells with intracytoplasmic lumina formation (H&E x 200); Also, positive reactivity of tumor cells for CD31 and CD34, and a high Ki67 index (CD31 × 400; CD34 × 400; Ki67 × 100)

The second chest CT showed no signs of pulmonary emboli or pneumonia. However, the pleural effusion had been reaccumulated. Moreover, the level of carbohydrate antigen 125 (CA-125) was elevated. Histopathological and IHC evaluation of the biopsy confirmed the diagnosis of PEHE, which was positive for CD31, CD34, CK, factor 8-R-antigen, and vimentin (Fig. 2).

**Fig. 3:**
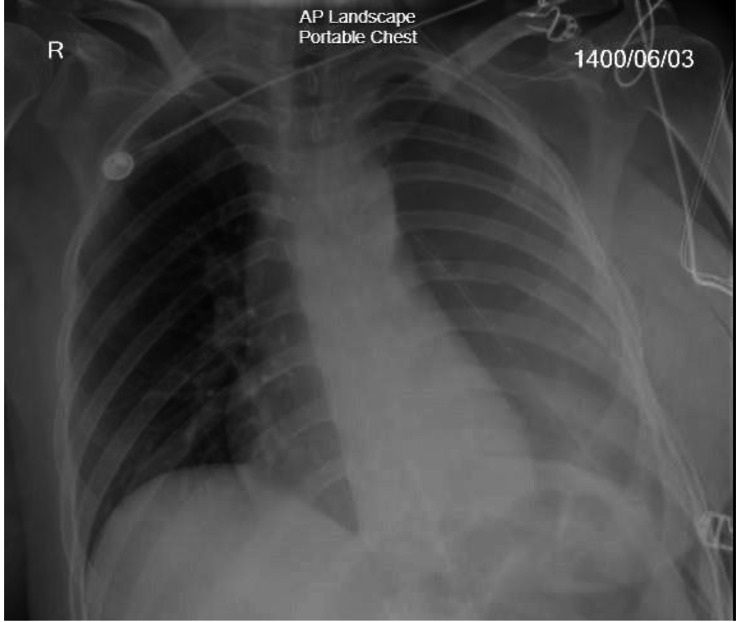
Post-operative chest radiography of a 39-year-old female patient with a diagnosis of pleural epithelioid hemangioendothelioma following extrapleural pneumonectomy

The patient was started on MAID regimen chemotherapy (Mesna, Adriamycin, Ifosfamide, Dacarbazine); However, despite the chemotherapy, the patient underwent extrapleural pneumonectomy duo to involvement of the adjacent lung, severe adhesions and thick pleura seven months later. The surgery was performed via left posterolateral thoracotomy, and the patient’s condition was acceptable after surgery (Fig. 3).

Unfortunately, during the disease course in November 2021, the patient developed multiple peritoneal metastasis, pericardial effusion, and ascites and died due to the complications of the disease. The result of abdominal paracentesis demonstrated a total count of 3400 cells, RBC 300, WBC 400 (lymphocyte 71% and neutrophil 29%), fluid sugar 89 mg/dl, protein 3.8 g/dl, albumin 2 g/dl, with a serum albumin of 3.2 g/dl.

## Discussion and conclusion

EHE can originate from many different anatomical sites but originating from the pleura is an extremely rare event [5]. PEHE in contrast to its counterpart, lung EHE which is usually an asymptomatic tumor commonly affecting young and middle-aged women with relatively better prognosis, almost always has a symptomatic aggressive clinical course but unlike our case, mostly affects older men [4, 8]. Based on a previous review by Rezvani et al. the mortality rate of pleural EHE is as high as 80% [6].

The etiology of EHE is believed to be due to either the t(1;3)(p36.3;q25) translocation in most case or the t(X;11)(p11;q22) translocation, which in turn result in WWTR1-CAMTA1 and YAP1-TFE3 fusion genes [8]. PEHE has also been related with exposure to asbestos or radiation but there is not enough evidence to prove it [9]. In our case, the patient didn’t have any significant family or past medical history or exposure to smoke and asbestos.

The prognosis of thoracic EHE can be quite variable on the basis of the site of origin and cannot be predicted based on the clinical and histopathologic grounds. Unfortunately, once the tumor is disseminated, it is hard to know for certain whether the primary site was pulmonary or pleural [6]. Differentiating EHE of lung from PEHE can be made by the presence or absence of nodular mass formation in subpleural lung parenchyma in radiologic examination and a histological examination on an intravascular, intraalveolar, and intrabronchial growth pattern. The differentiation between PEHE and diffuse pleural carcinomatosis or mesothelioma is also of importance and must be given careful consideration due to their similar radiologic appearances [5]. Like our case, suspicion must be confirmed using immunohistochemistry which are positive for Friend leukemia integration 1 transcription factor (Fli-1), CD31 and CD34. Together, these tests are highly specific and sensitive for EHE [10].

There are no standard treatments for PEHE yet. Using various chemotherapeutic agents, radiotherapy, surgery and a combination of these treatment methods as curative therapies in PEHE patients have failed to show promising results. Complete surgical resection of the nodules with follow up is the treatment of choice in solitary or limited number of lesions but a complete surgical resection is usually not possible in pleural EHE [5, 8]. Yu et al. suggested that radical resection should only be only considered if PEHE is locally extended [11]; However, extrapleural pneumonectomy was performed as the first line of treatment in 3 recently published cases with extensive tumor involvement (Table 1) [12–14]. In one case, the patient died after 3.5 months due to tumor recurrence, but in the other 2 cases desirable outcomes have been reported in which patients recovered well after the surgery. In our case, we also opted for a extrapleural pneumonectomy after poor response to chemotherapy and the extensive tumor involvement. Nevertheless, Further studies are needed to assess the effect of aggressive resection on survival of the patients.

**Table 1:** A review of PEHE cases who underwent surgery

Author; year	Age & sex	Initial complaint	Surgery	Adjuvant therapy	Survival in month
Crotty et al., 2000 [15]	Four patients, All male 55-71Y/O	Chest pain (3/4) Dyspnea (3/4) Productive cough & Fever (1/4) Weight loss (1/4)	Thoracotomy and pleural decortication (3/4) Video-assisted thoracoscopic pleural biopsy (1/4)	Not specified	Average 10 month
Al-Shraim et al., 2005 [16]	51 M	Dry cough and shortness of breath	Decortication & resection of the pleural tumor	Interferon α	> 24
Saqi et al., 2007 [17]	37 M	Dyspnea and pleuritic chest pain	Pleural decortication	A single cycle of carboplatin, etoposide, and avastin	2
Lee et al., 2008 [5]	31 F	Bilateral upper back pain	Thoracoscopic wedge resection of the right lower lobe of the lung	Adriamycin then switched to Mesna-Doxorubicin- Ifosfamide-Dacarbazine (MAID) + radiotherapy of spinal lesions	10
Chou et al., 2011 [18]	42 M	Chest pain and productive cough	Pleurectomy	Chemotherapy and radiotherapy after 5 month due to metastatic bony and spine lesions	> 14
	27 M	Dry cough, hoarseness and chest tenderness	Pleurectomy at 5 month	Radiotherapy and then doxorubicin and cisplatin at 5 month	18
Kim et al., 2011 [19]	46 F	Right-sided chest discomfort and cough	Decortication and visceral pleurectomy followed by complete pleurectomy and cytologic reduction to minimal disease	Carboplatin and etoposide	23
Lazarus et al., 2011 [20]	42 M	Cough, dyspnea, right sided back pain	Video-assisted thoracoscopic surgery (VATS), almost complete pleurectomy	Taxol and Bevacizumab	8
	42 M	Cough, low-grade fever	Decortication	Carboplatin, etoposide, and bevacizumab	6
Yu et al., 2013 [11]	39 F	Progressive dyspnea	Radical resection of the mass and left pericardium	Carboplatine and etoposide	No evidence of recurrence at 14 month
Ha et al., 2014 [21]	71 M	Cough, dyspnea, fatigue and poor oral intake	Wedge resection	Scheduled for chemotherapy	Not specified
Apolinário et al., 2016 [22]	47 F	Chest Pain and dyspnea	Pulmonary decortication and parietal pleurectomy	Doxorubicin, one cycle before death	6
Takenaka et al. 2020 [14]	62 M	Right chest pain and dyspnea on exertion	Extrapleural pneumonectomy due to extended disease	Pazopanib after recurrence in one month	3.5
Jebastin Thangaiah et al., 2021 [23]	39 F	Chest and back pain	Left lower lobe wedge resection	Clinical trial with Trametinib	> 15
Lavacchi et al., 2021 [13]	53 M	Chronic left hemithorax pain	Extrapleural pneumonectomy	Not specified	No radiological evidence of recurrent disease after 8 month
Hsu et al., 2022 [12]	35 M	Chronic non-productive cough	Extrapleural pneumonectomy	Radiation therapy after the surgery	Not concluded
Al-Nafisi et al., 2023 [24]	58 M	Incidental diagnosis in a lower back pain work up	VATS and pleural mass resection	Constant follow-up due to comorbidities	Not concluded
Pathak et al., 2023 [25]	73 F	Dyspnea, cough, and pleuritic Chest pain	VATS with decortication	Chemo/radiation therapy was started after the study	Not concluded
Our case	39 F	Progressive dyspnea and left shoulder discomfort	Extrapleural pneumonectomy at 7 month	Mesna, Adriamycin, Ifosfamide, Dacarbazine (MAID) regimen	10

**VATS**: Video-assisted thoracoscopic surgery.

Chemotherapy therapy regimens are quit variable in different studies. Chemotherapy with etoposide and carboplatin as a part of treatment showed longer survival rate in some cases and in one case, MAID regimen plus palliative radiotherapy of the spine metastasis showed improvement in symptoms and survival of 10 months [5, 6]. The MAID regimen and the following surgery used in our patient also resulted in a 10-month survival.

In conclusion, differentiation of PEHE from the pulmonary counterparts is an issue of concern as prognosis and treatment are different. Clinical and radiographic similarities between PEHE and mesothelioma and diffuse pleural carcinomatosis makes the definite diagnosis challenging. Once EHE is suspected through histology, it can be confirmed with immunohistochemistry. In this case, biopsy of parietal pleura was sent to the laboratory and the diagnosis of PEHE was confirmed. Chemotherapy followed by an extrapleural pneumonectomy is used as the treatment in this case; However, based on available data, the standard treatment still remains a question.

## Data Availability

All data regarding this case has been reported in the manuscript. Please write to the corresponding author if you are interested in any additional data.

## Abbreviations

CT: Computed tomography
EHE: Epithelioid hemangioendothelioma
PEHE: Pleural Epithelioid hemangioendothelioma
RBC: Red Blood cell
WBC: White blood cell
VATS: Video-assisted thoracoscopic surgery
